# Baseline representativeness of patients in clinics enrolled in the PRimary care Opioid Use Disorders treatment (PROUD) trial: comparison of trial and non-trial clinics in the same health systems

**DOI:** 10.1186/s12913-022-08915-1

**Published:** 2022-12-29

**Authors:** Paige D Wartko, Hongxiang Qiu, Abisola E. Idu, Onchee Yu, Jennifer McCormack, Abigail G. Matthews, Jennifer F. Bobb, Andrew J. Saxon, Cynthia I. Campbell, David Liu, Jordan M. Braciszewski, Sean M. Murphy, Rachael P. Burganowski, Mark T. Murphy, Viviana E. Horigian, Leah K. Hamilton, Amy K. Lee, Denise M. Boudreau, Katharine A. Bradley

**Affiliations:** 1 Kaiser Permanente Washington Health Research Institute, 1730 Minor Ave, Ste 1600, Seattle, WA 98101 United States; 2 Department of Biostatistics, University of Washington, 1705 NE Pacific Street, Seattle, WA 98195 United States; 3 Department of Statistics and Data Science, University of Pennsylvania, 3451 Walnut St Philadelphia, Philadelphia, PA 19104 United States; 4 The Emmes Company, 401 N Washington St #700, Rockville, MD 20850 United States; 5 Center of Excellence in Substance Addiction Treatment and Education, VA Puget Sound Health Care System, 1660 S Columbian Way, Seattle, WA 98108 United States; 6 Kaiser Permanente Northern California Division of Research, 2000 Broadway, Oakland, CA 94612 United States; 7 National Institute on Drug Abuse Center for Clinical Trials Network, Three White Flint North, 11601 Landsdown Street, North Bethesda, MD 20852 United States; 8 Henry Ford Health, One Ford Place, Suite 3A, Detroit, MI 48202 United States; 9 Department of Population Health Sciences, Weill Cornell Medical College, 1300 York Ave, New York, NY 10065 United States; 10 MultiCare Health System, 315 Martin Luther King Jr. Way, Tacoma, WA 98415 United States; 11 Department of Public Health Sciences, Miller School of Medicine, University of Miami, 1120 NW 14th St, CRB 919, Miami, FL 33136 United States; 12 Genentech, 1 DNA Way, South San Francisco, CA 94080 United States

**Keywords:** Buprenorphine, Medication, Nurse care manager, Opioid use disorder, Implementation trial, Primary care

## Abstract

**Background:**

Pragmatic primary care trials aim to test interventions in “real world” health care settings, but clinics willing and able to participate in trials may not be representative of typical clinics. This analysis compared patients in participating and non-participating clinics from the same health systems at baseline in the PRimary care Opioid Use Disorders treatment (PROUD) trial.

**Methods:**

This observational analysis relied on secondary electronic health record and administrative claims data in 5 of 6 health systems in the PROUD trial. The sample included patients 16–90 years at an eligible primary care visit in the 3 years before randomization. Each system contributed 2 randomized PROUD trial clinics and 4 similarly sized non-trial clinics. We summarized patient characteristics in trial and non-trial clinics in the 2 years before randomization (“baseline”). Using mixed-effect regression models, we compared trial and non-trial clinics on a baseline measure of the primary trial outcome (clinic-level patient-years of opioid use disorder (OUD) treatment, scaled per 10,000 primary care patients seen) and a baseline measure of the secondary trial outcome (patient-level days of acute care utilization among patients with OUD).

**Results:**

Patients were generally similar between the 10 trial clinics (*n* = 248,436) and 20 non-trial clinics (*n* = 341,130), although trial clinics’ patients were slightly younger, more likely to be Hispanic/Latinx, less likely to be white, more likely to have Medicaid/subsidized insurance, and lived in less wealthy neighborhoods. Baseline outcomes did not differ between trial and non-trial clinics: trial clinics had 1.0 more patient-year of OUD treatment per 10,000 patients (95% CI: − 2.9, 5.0) and a 4% higher rate of days of acute care utilization than non-trial clinics (rate ratio: 1.04; 95% CI: 0.76, 1.42).

**Conclusions:**

trial clinics and non-trial clinics were similar regarding most measured patient characteristics, and no differences were observed in baseline measures of trial primary and secondary outcomes. These findings suggest trial clinics were representative of comparably sized clinics within the same health systems. Although results do not reflect generalizability more broadly, this study illustrates an approach to assess representativeness of clinics in future pragmatic primary care trials.

**Supplementary Information:**

The online version contains supplementary material available at 10.1186/s12913-022-08915-1.

## Contributions to the literature

- Pragmatic trials randomly assign primary care clinics to test interventions in “real world” settings, but primary care clinics that participate in pragmatic trials may not be representative of typical primary care clinics in the study health systems.
- We compared similarly sized trial and non-trial primary care clinics in the PROUD trial and found that they had largely similar patient populations and baseline measures of trial outcomes.
- Findings suggest patients in the PROUD trial clinics were representative of those in other clinics within the same health systems. This type of analysis could help to assess generalizability of results in future trials.

## Background

Pragmatic and implementation trials in primary care settings aim to test interventions in the “real world” [1]. However, primary care clinics that are willing and able to participate in these trials may not be representative of typical clinics in their health systems. If participating primary care clinics differ meaningfully from non-participating clinics in ways that impact the effect of the intervention, generalizability may be limited. The extension of Consolidated Standards of Reporting trials (CONSORT) guidelines for pragmatic trials recommends reporting the number of clinics approached and the reasons they declined, in order to indirectly assess the potential for recruitment of a biased sample of clinics [2]. Despite awareness of this issue and prior assessments comparing *individuals* participating in trials with eligible non-participants [3–5], we do not know of any prior cluster-randomized health care trials that have conducted such analyses comparing *clinics* that do and do not participate.

This analysis used baseline data to compare patient characteristics and baseline measures of trial outcomes in participating and non-participating primary care clinics in the PRimary care Opioid Use Disorders treatment (PROUD) trial (NCT03407638), a pragmatic, hybrid type III cluster-randomized implementation trial. The PROUD trial aimed to evaluate whether implementation of the Massachusetts Model [6] of office-based addiction treatment (OBAT) for management of opioid use disorder (OUD) in primary care increased treatment with buprenorphine or extended-release injectable naltrexone (XR-NTX). Secondarily, the PROUD trial evaluated whether the intervention decreased acute care utilization among patients with OUD prior to randomization. A full study protocol for the PROUD trial was previously published [7]. Six diverse health systems participated in the trial, and each identified two primary care clinics to be randomized to intervention or usual care control (stratified by health system).

This study compared primary care clinics chosen by health system leaders to participate in the PROUD trial (“trial clinics” hereafter) to similarly sized, randomly chosen primary care clinics within the same health systems at baseline (“non-trial clinics” hereafter), to evaluate the representativeness of trial clinics. This evaluation had 3 objectives. The 1st objective was to descriptively compare baseline characteristics of patients seen in trial and non-trial primary care clinics. The 2nd objective was to evaluate whether medication treatment for OUD differed at baseline between patients in trial and non-trial clinics; this was a baseline measure of the primary (implementation) outcome of the PROUD trial. The 3rd objective was to evaluate whether acute care utilization differed at baseline between patients with OUD in the trial and non-trial clinics; this was a baseline measure of the main secondary (effectiveness) outcome of the PROUD trial.

## Methods

### Study samples and data

To participate in the PROUD trial, site lead investigators and their health systems were required to identify 2 primary care clinics, each with ≥10,000 patients seen annually. A “clinic” could be a cluster of 2 to 3 smaller clinics that were geographically close enough to each other to share a nurse care manager if randomized to the intervention. We required the 2 trial clinics within the same health system to have largely separate populations so that patient cross-over between trial clinics (intervention and usual care control) was unlikely. Health system and clinic leaders provided letters of support agreeing to participate in the trial, which included integrating a nurse care manager into the clinic and having at least 3 primary care providers who were willing to obtain Drug Enforcement Agency (DEA) waivers to prescribe buprenorphine for OUD in the intervention clinic. We required the health systems to be able to provide secondary data for PROUD trial outcome measures, which we evaluated during the developmental phase of PROUD (Phase 1). Other infrastructure required more recently for implementation of the Massachusetts OBAT model for Medicaid (e.g., counseling) was not required for the PROUD trial [8]. No stipulations were made regarding primary care teams’ interest or motivation regarding treatment of OUD. Because this intervention required buy-in from clinical leaders in the health system, it was not feasible to randomly select clinics for the trial from all primary care clinics in each health system.

We enrolled 6 geographically diverse health systems in the PROUD trial, but one health system did not meet our requirement for this analysis of having at least 4 non-trial primary care clinics without a program of exemplar medication treatment for OUD at baseline and seeing ≥7500 patients annually. (There were insufficient numbers of clinics with ≥10,000 patients, as had been required for trial clinics.) Thus, for the present analyses, we only included 5 health systems (in New York, Michigan, Texas, and 2 in Washington). Preliminary estimates provided by these 5 health systems revealed a mean of 22 total primary care clinics in each health system (standard deviation [SD]: 4.5), a mean of 9872 patients seen per year in each clinic (SD: 9766.8), and a mean of 10 clinics in each health system (SD: 4.4) that were not trial or exemplar clinics and had ≥7500 patients. All but one of these 5 health systems had more than 4 such non-trial primary care clinics, for which a study biostatistician randomly selected which clinics to use for these analyses.

The sample for the OUD treatment outcome and secondary outcomes related to OUD diagnosis and treatment included patients seen in trial or non-trial clinics who were age 16–90 years and had a primary care visit during the 3 years prior to the randomization date, except for one health system with an eligibility period of only 2.8 years prior to randomization due to an electronic health record system change. Randomization occurred on 2/28/2018 for 4 health systems and 8/29/2018 for the other health system due to a delay in the data use agreement, and health systems were notified of clinics’ assignments on 2/28/2018 and 8/31/2018, respectively (jointly referred to as “randomization date” hereafter). The sample for the acute care utilization outcome was the same as for the OUD treatment outcome, with the additional requirement that patients had to have a documented OUD diagnosis (Supplemental Table 1) in the 3 years prior to randomization.

All data for this study were secondary electronic health record data, including data from administrative sources and insurance claims, which included patient characteristics and OUD treatment-related outcomes available in trial and non-trial primary care clinics. Other than number of primary care providers and buprenorphine prescribers, we did not collect clinic measures (such as infrastructure, services, staffing, culture, or attitudes about OUD treatment) for the non-trial clinics, so such measures were not included in this analysis.

A single Institutional Review Board (IRB), Advarra, approved the study with all health systems ceding, including providing waivers of consent and Health Insurance Portability and Accountability Act (HIPAA) authorization, consistent with US regulations [9]. Approval from the IRB and subsequent data use agreements allowed health system programmers to obtain and share limited datasets (as defined by HIPAA) with the lead site after data cleaning locally [10]. The datasets included dates and zip codes but no other data considered identifiable by HIPAA. The purpose of only sharing limited datasets is to protect patient privacy. Each health system securely transferred their limited datasets to the lead site where data were reviewed by the lead study data and analytics team who had expertise in using electronic health record data for research. This team included programmers, biostatisticians, a pharmacoepidemiologist, and a pharmacist-pharmacoepidemiologist, who consulted with health system programmers, Site Lead Investigators and the study Principal Investigator (a physician-researcher) as needed to address any questions. When necessary to correct identified issues, data were re-extracted by programmers at the health systems. This multi-step, iterative data quality checking process served to assure data integrity for accurate scientific results.

### Measures

#### Measure timing

**Fig. 1:**
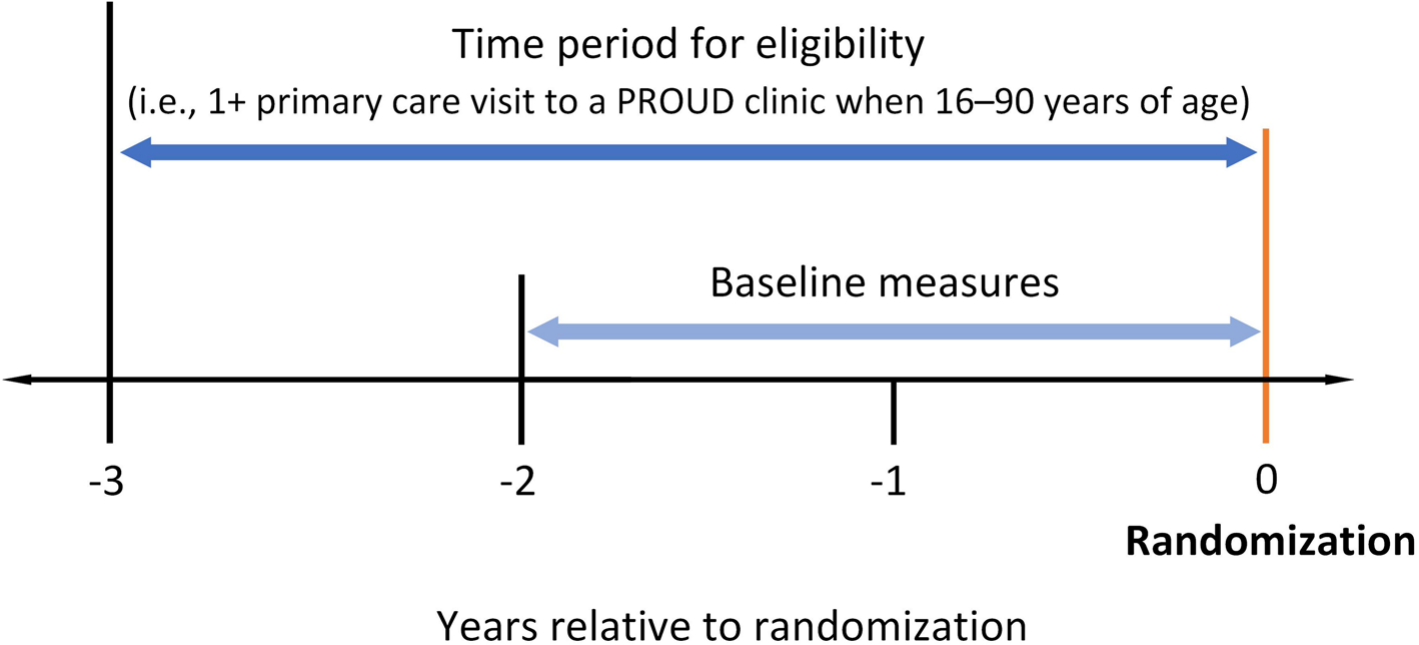
Legend. Study timeline

While the time period for eligibility was the 3 years prior to randomization, the measures were assessed during the 2 years prior to randomization (hereafter, “baseline”), as illustrated in Fig. 1.

#### Characteristics of patients and number of clinic providers

We described characteristics at the patient-level, including age (at the time of randomization), sex, race, and ethnicity. As with all other data in this analysis, race and ethnicity information was ascertained from electronic health records. If a patient was Hispanic or Latinx, they were presented in the Hispanic or Latinx category, and not in a racial group (Asian, Black or African American, American Indian or Alaska Native, Native Hawaiian or Pacific Islander, white, multiple race, other race). Our understanding was that “other race” was recorded in the electronic health record when a person did not identify as belonging to any of the previously listed racial groups (and was not Hispanic or Latinx). In this paper, we based terminology for race and ethnicity on updated recommendations in the American Medical Association’s style manual [11]. We reported insurance status from the most recent study year prior to randomization in which the person had an eligible primary care visit. Insurance categories included Medicare, Medicaid/subsidized, otherwise insured, or uninsured. If patients had evidence of multiple insurance types over the year, we included them in multiple categories.

We defined mental health, substance use, and other relevant health conditions, including hepatitis C virus (HCV) infection, human immunodeficiency virus (HIV) infection, and non-cancer pain (diagnoses in Mayhew, et al. [12]), as having at least one ICD-10 diagnosis code for that condition in the baseline period. We calculated the Elixhauser Comorbidity Index over the year prior to randomization [13]. We presented neighborhood-level socioeconomic status measures for each patient from the 2014–2018 5-year estimates from the U.S. Census Bureau American Community Survey [14] based on patients’ zip code at randomization date, or the value before randomization date that was closest to it.

Additionally, we described the clinics’ number of primary care providers and buprenorphine prescribers.

#### OUD treatment and related outcomes

The PROUD trial’s primary outcome is the number of patient-years of OUD medication treatment at baseline among all eligible primary care patients. For the present study, we created a baseline measure of this outcome: days of OUD treatment and related measures at the clinic-level, scaled by 10,000 patients seen in the clinic during the baseline period (to account for variability in clinic size). We defined treatment for OUD as having a medication order or procedure code (Supplemental Table 2) for buprenorphine formulations approved by the Food and Drug Administration for OUD (sublingual tablet or film, buccal film, subdermal implants, subcutaneous extended-release injection) or XR-NTX, although we only counted XR-NTX if the patient had at least 2 instances of an OUD or opioid overdose diagnostic code (Supplemental Tables 1 and 3).

We calculated other baseline measures reflecting OUD treatment in primary care, as planned for the trial [7], including the number of patients with a documented OUD diagnosis (Supplemental Table 1), initiating OUD treatment (treatment during the 2-year baseline period but not in the year prior), and any OUD treatment during the baseline period.

To further assess OUD treatment, we included two secondary measures: a measure of the number of patients with ≥6 months of treatment and a measure of the number of patients with ≥80% days covered. To allow 6 months of treatment to be observable and enough time for 80% of days covered to be meaningful, we restricted to patients who entered the cohort ≥6 months before randomization date. This restriction also applied to the denominator (number of patients seen in the clinic). To provide context for these measures, we also calculated a measure of number of patients with an OUD diagnosis restricted to patients who entered the cohort ≥6 months before randomization date.

#### Acute care utilization outcomes

The PROUD trial’s main secondary effectiveness outcome is days of acute care utilization among patients with OUD documented at baseline. To correspond with this secondary outcome [7], we calculated baseline patient-level acute care utilization, among patients with a documented OUD diagnosis in the 3 years prior to randomization. This measure included 3 types of acute care utilization (urgent care, emergency department, and inpatient health care utilization), and additional measures assessed urgent care/emergency department and inpatient acute care utilization separately.

### Statistical analyses

We used summary statistics to describe patient characteristics at the patient-level separately for trial and non-trial clinics, both in all primary care patients and in the subgroup with OUD. We did not test differences in characteristics between patients in trial and non-trial clinics because this large sample size would likely yield many statistically significant differences. Instead, we calculated standardized mean differences (SMDs), which compared the difference in means (or prevalence) of a characteristic in units of the pooled standard deviation. This measure of difference is less influenced by sample size and enables comparison of the relative balance of variables even if they are not in the same units [15]. While there is no universally accepted cut point for meaningful imbalance, SMDs of < 0.10 are typically considered unimportant [16], and we used this as an approximate guideline in interpreting these results.

We also calculated summary statistics and SMDs for clinic-level number of primary care providers and buprenorphine prescribers.

For each clinic-level outcome of OUD treatment implementation, we fit a mixed-effect linear regression model that included a health system-specific random intercept to account for correlation of clinics within the same health system. We used the same model type for the OUD treatment-related secondary outcomes restricting to patients who entered the cohort ≥6 months before randomization date. This approach followed the statistical analysis plan for the trial analyses of OUD treatment outcomes. The models estimated mean differences (MDs) and 95% confidence intervals (CIs) comparing trial and non-trial clinics.

For patient-level outcomes of acute care utilization measures among those with a documented OUD diagnosis, we fit a mixed-effect Poisson regression model that included clinic-specific random intercepts to account for correlation of patients within the same clinic. This approach followed that of the statistical analysis plan for the trial analyses of acute care utilization outcomes. The models estimated rate ratios (RRs) and 95% CIs comparing trial and non-trial clinics.

Because the purpose of this analysis was to evaluate whether trial and non-trial clinics differed, we decided a priori to conduct all analyses unadjusted to avoid adjusting away the differences we were interested in observing.

Regarding missing data, our outcomes were defined by the presence of a medication order, procedure code, diagnosis code, or acute care visit, such that it was not possible to distinguish between a patient truly not having the outcome or our data not capturing an outcome they had. For some of the descriptive patient characteristics, we expected all patients to have a value, so we knew when patients were missing that information (e.g., race and ethnicity). We reported the small percentage of patients missing values for these variables in Table 1 footnotes, but it did not impact statistical analyses because we used unadjusted models that did not include patient characteristics.

We conducted analyses in the Statistical Analysis System (version 9.4; SAS Institute, Cary, NC) and R (version 3.6.3).

## Results

### Characteristics of all eligible patients and number of clinic providers

There were 589,566 eligible patients seen in primary care in the 3 years before randomization across the five health systems, with 248,436 from the 10 PROUD trial clinics (average size: 24,844 patients) and 341,130 from the 20 non-trial clinics (average size: 17,057 patients) (data not tabled).

As presented in Table 1, at baseline, patients seen in clinics enrolled in the trial were generally similar to those in clinics not enrolled in the trial, with some exceptions: patients in trial clinics were slightly younger (mean age: 48.5 years [standard deviation (SD): 17.7] vs. 50.2 years [SD: 18.1], SMD: 0.096), more likely to be Hispanic or Latinx (27.4% vs. 21.5%, SMD: 0.146), less likely to be white (35.6% vs. 41.9%, SMD: 0.129), less likely to have Medicare insurance (19.0% vs. 23.7%, SMD: 0.116), and more likely to have Medicaid/subsidized insurance (39.9% vs. 33.0%, SMD: 0.145). Patients in trial clinics, as compared with those in non-trial clinics, lived in neighborhoods with a lower median household income ($54,000 vs. $60,000, SMD: 0.220), a higher proportion of residents living below the federal poverty level (16.7% vs. 13.3%, SMD: 0.277), and a higher median rent to income ratio for neighborhood residents (31.9% vs. 30.6%, SMD: 0.276).

**Table 1:** Characteristics among patients seen in primary care in the 3 years before randomization

*Characteristics*	All patients *(Eligible population for OUD treatment analysis)* *N = 589,566*					Patients with documented OUD diagnosis *(Eligible population for acute care utilization analysis)* *N = 4658*				
	*10 PROUD trial clinics* *No. patients =* 248,436		*20 non-trial clinics* *No. patients =* 341,130		*SMD*	*10 PROUD trial clinics* *No. patients =* 1935		*20 non-trial clinics* *No. patients =* 2723		*SMD*
Age in years										
Mean (SD)	48.5	(17.7)	50.2	(18.1)	0.096	48.9	(15.9)	50.5	(15.8)	0.095
n (%)										
16–17	3019	(1.2)	4326	(1.3)		0	(0.0)	2	(0.1)	
18–24	22,039	(8.9)	28,667	(8.4)		99	(5.1)	104	(3.8)	
25–44	80,456	(32.4)	98,247	(28.8)		672	(34.7)	888	(32.6)	
45–64	93,917	(37.8)	130,725	(38.3)		836	(43.2)	1213	(44.5)	
65–74	30,424	(12.2)	48,160	(14.1)		225	(11.6)	368	(13.5)	
≥ 75	18,581	(7.5)	31,005	(9.1)		103	(5.3)	148	(5.4)	
Female,^a^ n (%)	152,932	(61.6)	201,992	(59.2)	0.048	1033	(53.4)	1486	(54.6)	0.024
Race and ethnicity,^b^ n (%)										
Hispanic or Latinx	68,085	(27.4)	73,476	(21.5)	0.146	281	(14.5)	294	(10.8)	0.113
Asian	13,903	(5.6)	14,600	(4.3)	0.064	26	(1.3)	32	(1.2)	0.015
Black or African American	51,797	(20.8)	78,027	(22.9)	0.048	324	(16.7)	445	(16.3)	0.009
American Indian or Alaska Native	1346	(0.5)	2467	(0.7)	0.023	38	(2.0)	52	(1.9)	0.003
Native Hawaiian or Pacific Islander	1605	(0.6)	1830	(0.5)	0.015	9	(0.5)	13	(0.5)	0.002
White	88,565	(35.6)	142,771	(41.9)	0.129	1130	(58.4)	1738	(63.8)	0.124
Multiple race	996	(0.4)	826	(0.2)	0.029	12	(0.6)	7	(0.3)	0.056
Other race	7269	(2.9)	8599	(2.5)	0.027	38	(2.0)	23	(0.8)	0.097
Insurance status,^c^ n (%)										
Medicare	47,212	(19.0)	80,829	(23.7)	0.116	583	(30.1)	897	(32.9)	0.061
Medicaid/subsidized	99,164	(39.9)	112,464	(33.0)	0.145	781	(40.4)	1085	(39.8)	0.011
Otherwise insured	121,731	(49.0)	176,618	(51.8)	0.057	844	(43.6)	1175	(43.2)	0.010
Uninsured	10,905	(4.4)	14,826	(4.3)	0.002	64	(3.3)	129	(4.7)	0.073
Number of primary care visits, median (IQR)	3	(1, 5)	2	(1, 5)	0.036	5	(2, 9)	5	(2, 9)	0.041
Documented mental health condition, n (%)										
Any of the following	56,865	(22.9)	83,729	(24.5)	0.039	1288	(66.6)	1781	(65.4)	0.024
Depression	34,789	(14.0)	50,575	(14.8)	0.023	929	(48.0)	1257	(46.2)	0.037
Anxiety	37,315	(15.0)	54,192	(15.9)	0.024	912	(47.1)	1291	(47.4)	0.006
Post-traumatic stress disorder	2960	(1.2)	4580	(1.3)	0.014	151	(7.8)	253	(9.3)	0.053
Attention deficit hyperactivity disorder	2818	(1.1)	4344	(1.3)	0.013	94	(4.9)	123	(4.5)	0.016
Eating disorder	511	(0.2)	836	(0.2)	0.008	10	(0.5)	19	(0.7)	0.023
Bipolar spectrum disorder	4215	(1.7)	6217	(1.8)	0.010	235	(12.1)	327	(12.0)	0.004
Schizophrenia/psychoses	2191	(0.9)	3722	(1.1)	0.021	76	(3.9)	163	(6.0)	0.095
Documented non-opioid SUD, n (%)										
Tobacco use disorder	21,423	(8.6)	32,435	(9.5)	0.031	797	(41.2)	1041	(38.2)	0.060
Alcohol use disorder	5411	(2.2)	9581	(2.8)	0.040	293	(15.1)	458	(16.8)	0.046
Other non-opioid SUD	4076	(1.6)	7187	(2.1)	0.034	556	(28.7)	743	(27.3)	0.032
Cannabis use disorder	2134	(0.9)	3344	(1.0)	0.013	175	(9.0)	214	(7.9)	0.043
Stimulant use disorder^d^	1184	(0.5)	2364	(0.7)	0.028	233	(12.0)	318	(11.7)	0.011
Other SUD^e^	1567	(0.6)	3170	(0.9)	0.034	366	(18.9)	538	(19.8)	0.021
Opioid overdose,^f^ n (%)	122	(< 0.1)	163	(< 0.1)	0.001	49	(2.5)	61	(2.2)	0.019
Non-opioid overdose,^g^ n (%)	133	(0.1)	210	(0.1)	0.003	22	(1.1)	33	(1.2)	0.007
Other documented chronic conditions, n (%)										
HCV	2363	(1.0)	4270	(1.3)	0.029	229	(11.8)	318	(11.7)	0.005
HIV	823	(0.3)	977	(0.3)	0.008	23	(1.2)	26	(1.0)	0.023
Non-cancer pain	160,394	(64.6)	221,195	(64.8)	0.006	1576	(81.4)	2206	(81.0)	0.011
Elixhauser Comorbidity Index, n (%)										
0	145,419	(58.5)	192,554	(56.4)	0.042	698	(36.1)	879	(32.3)	0.080
1	38,302	(15.4)	52,017	(15.2)	0.005	222	(11.5)	317	(11.6)	0.005
≥ 2	64,715	(26.0)	96,559	(28.3)	0.051	1015	(52.5)	1527	(56.1)	0.073
Housing instability diagnosis code, n (%)	1574	(0.6)	3380	(1.0)	0.040	77	(4.0)	161	(5.9)	0.089
Homelessness diagnosis code, n (%)	701	(0.3)	2437	(0.7)	0.061	57	(2.9)	136	(5.0)	0.105
Neighborhood-level household SES										
Median household income,^h^ median (IQR)	54K	(39K, 76K)	60K	(47K, 76K)	0.220	58K	(43K, 77K)	62K	(51K, 76K)	0.157
Percent below FPL, median (IQR)	16.7	(8.7, 27.9)	13.3	(8.9, 20.6)	0.277	14.5	(7.8, 23.1)	12.7	(8.9, 19.2)	0.238
Rent as percent of income, median (IQR)	31.9	(28.9, 35.0)	30.6	(28.0, 33.1)	0.276	31.1	(29.1, 34.1)	30.6	(28.3, 32.7)	0.282
Percent unemployed, median (IQR)	6.2	(4.9, 8.6)	6.6	(5.1, 8.6)	0.011	6.2	(4.6, 8.4)	6.5	(5.1, 8.0)	0.081

*OUD* Opioid use disorder, *SMD* Standardized mean difference, *SD* Standard deviation, *IQR* Interquartile range, *SUD* Substance use disorder, *HCV* Hepatitis C virus, *HIV* Human immunodeficiency virus, *SES *Socioeconomic status, *FPL* Federal poverty level

^a^Patients who were not identified as female were identified as male, except for 3 patients (< 0.1%) missing this information at PROUD trial clinics, and 5 patients (< 0.1%) missing this information at non-trial clinics. None of the patients with a documented OUD diagnosis were missing this information

^b^At PROUD trial clinics, 14,870 patients (6.0%) were missing both race and ethnicity data, and at non-trial clinics, 18,534 patients (5.4%) were missing this information. Among patients with OUD, 77 (4.0%) were missing both race and ethnicity data at trial clinics, and 119 (4.4%) were missing this information at non-trial clinics

^c^At PROUD trial clinics, 2715 patients (1.1%) were missing insurance information, and at non-trial clinics 4026 patients (1.2%) were missing this information. Among patients with OUD, 14 (0.7%) were missing insurance information at trial clinics, and 19 (0.7%) were missing this information at non-trial clinics

^d^Cocaine, amphetamine, and other stimulant use disorders

^e^Sedative, hypnotic, anxiolytic, hallucinogen, inhalant, and any other psychoactive substance use disorders

^f^Fatal and non-fatal opioid overdose, follow-up after overdose, or sequelae

^g^Fatal and non-fatal alcohol, cocaine or other stimulant, cannabis, and other non-opioid overdose, follow-up after overdose, or sequelae

^h^Presented in US dollars, where K = $1000

The average number of primary care providers was greater in trial clinics, at 36.8 (SD: 19.1), compared with 30.0 at non-trial clinics (SD: 33.4; SMD: 0.252), but the average number of buprenorphine prescribers was essentially the same in trial and non-trial clinics (1.6 [SD: 1.6] vs. 1.7 [SD: 2.4], SMD: 0.049) (data not tabled).

### OUD treatment and related outcomes

At baseline, differences in treatment for OUD and related outcomes were small in magnitude and not statistically significant comparing the 10 PROUD trial clinics and 20 non-trial clinics (Table 2). On average, 72.4 versus 66.2 patients had documented OUD diagnoses per 10,000 patients seen in trial versus non-trial clinics, respectively (MD: 6.2 patients per 10,000, 95% CI: −14.7 to 27.1). Per 10,000 patients seen, 8.3 versus 6.1 patients initiated OUD treatment in trial and non-trial clinics, respectively (MD: 2.2 patients treated per 10,000, 95% CI: −1.6 to 6.0), while 12.6 versus 9.7 patients had any OUD treatment per 10,000 patients seen in trial and non-trial clinics, respectively (MD: 2.9 patients treated per 10,000, 95% CI: −2.3 to 8.1). For the baseline measure of the primary PROUD trial outcome, we observed an average of 8.0 versus 7.0 patient-years of OUD treatment per 10,000 patients in trial and non-trial clinics, respectively (MD: 1.0 patient-year per 10,000 patients, 95% CI: −2.9 to 5.0).

**Table 2:** OUD diagnosis and medication treatment at the clinic-level in the 2 years before randomization date

Measures scaled per 10,000 primary care patients seen in the clinic in the 2 years prior to randomization	10 PROUD trial clinics Mean (SD) across clinics	20 non-trial clinics Mean (SD) across clinics	Mean difference (95% CI) from mixed-effect linear model
*Number of patients with:*			
Documented OUD diagnosis	72.4 (42.1)	66.2 (33.4)	6.2 (− 14.7 to 27.1)
Initiation of OUD treatment	8.3 (7.0)	6.1 (4.3)	2.2 (−1.6 to 6.0)
Any OUD treatment	12.6 (9.5)	9.7 (6.6)	2.9 (−2.3 to 8.1)
Patient-years of OUD treatment^a^	8.0 (5.2)	7.0 (5.4)	1.0 (−2.9 to 5.0)
*Restricting to the sample of patients with at least 6 months of observation in study period*, *number of patients with:*			
Documented OUD diagnosis	75.5 (45.9)	65.6 (35.7)	9.9 (−12.0 to 31.7)
≥80% of days covered by OUD treatment^b^	3.5 (2.4)	3.1 (2.4)	0.3 (−1.5 to 2.1)
≥6 months covered by OUD treatment^c^	6.3 (4.8)	5.0 (4.2)	1.4 (−1.4 to 4.1)

*OUD* Opioid use disorder, *SD* standard deviation, *XR-NTX* extended-release injectable naltrexone

^a^Primary PROUD trial outcome

^b^Calculated as percentage of days from first OUD medication treatment occurring ≥6 months before randomization date to enable ≥6 months of follow-up to be observed after the first documented treatment, smoothing over gaps ≤14 days between a buprenorphine end date and XR-NTX start date

^c^Calculated treatment starting ≥6 months before randomization date, smoothing over gaps of ≤7 days or gaps of ≤14 days between a buprenorphine end date and XR-NTX start date

Restricting the sample to patients with ≥6 months of observation produced similar results (Table 2). There were no significant differences between trial and non-trial clinics in the number of patients per 10,000 with: documented OUD diagnoses (MD: 9.9 more patients per 10,000, 95% CI: −12.0 to 31.7), ≥80% of days covered by OUD treatment (MD: 0.3 patients per 10,000, 95% CI: −1.5 to 2.1), or ≥ 6 months covered by OUD treatment (MD: 1.4 patients per 10,000, 95% CI: −1.4 to 4.1).

### Characteristics of patients with OUD

There were 4658 eligible primary care patients with documented OUD in the 3 years before randomization, including 1935 at trial clinics and 2723 at non-trial clinics (Table 1). As in the overall sample, trial clinic patients with documented OUD were slightly younger (mean age: 48.9 years [SD: 15.9] vs. 50.5 years [SD: 15.8], SMD: 0.095), more likely to be Hispanic or Latinx (14.5% vs. 10.8%, SMD: 0.113), and less likely to be white (58.4% vs. 63.8%, SMD: 0.124) than patients with documented OUD in non-trial clinics. Patients with OUD in the trial clinics were potentially less likely than those in the non-trial clinics to have schizophrenia or other psychoses (3.9% vs. 6.0%, SMD: 0.095). Patients with documented OUD in the trial clinics, as compared with patients with documented OUD in non-trial clinics, were less likely to have a homelessness diagnosis code (2.9% vs. 5.0%, SMD: 0.105) but, similar to the overall sample, lived in neighborhoods with a lower median household income ($58,000 vs. $62,000, SMD: 0.157), a higher proportion of residents below the federal poverty level (14.5% vs. 12.7%, SMD: 0.238), and a higher median rent to income ratio for neighborhood residents than patients in non-trial clinics (31.1% vs. 30.6%, SMD: 0.282).

### Acute care utilization outcomes

Differences in patient-level measures of acute care utilization at baseline among patients with a documented OUD diagnosis in the 10 trial clinics and 20 non-trial clinics were small and not statistically significant (Table 3). Specifically, patients with documented OUD in trial and non-trial clinics had an average of 4.7 versus 4.6 days per year with any acute care utilization, respectively (RR: 1.04; 95% CI: 0.76 to 1.42), reflecting 1.4 versus 1.5 days of urgent care or emergency department utilization (RR: 1.04; 95% CI: 0.74 to 1.48) and 3.3 versus 3.2 days hospitalized, respectively (RR: 1.00; 95% CI: 0.68 to 1.46).

**Table 3:** Patient-level acute care utilization 2 years before randomization, among primary care patients with OUD^a^

Annual days per patient of:	Patients with documented OUD diagnosis^a^		
	10 PROUD trial clinics	20 non-trial clinics	Rate ratio (95% CI) from mixed-effect Poisson model
Any urgent care, emergency department, or inpatient care utilization^b^			
Median (IQR)	1.5 (0.0 to 5.0)	1.0 (0.0 to 4.5)	
Mean (SD)	4.7 (10.0)	4.6 (9.2)	1.04 (0.76 to 1.42)
Urgent care or emergency department utilization^c^			
Median (IQR)	0.5 (0.0 to 1.5)	0.5 (0.0 to 2.0)	
Mean (SD)	1.4 (2.6)	1.5 (2.5)	1.04 (0.74 to 1.48)
Inpatient care utilization (hospitalization)			
Median (IQR)	0.0 (0.0 to 2.5)	0.0 (0.0 to 2.5)	
Mean (SD)	3.3 (9.1)	3.2 (8.1)	1.00 (0.68 to 1.46)

*OUD* Opioid use disorder, *IQR* Interquartile range, *SD* Standard deviation

^a^Defined as a documented OUD diagnosis in the 3 years before randomization

^b^Secondary PROUD trial outcome

^c^We assumed urgent care and emergency department visits to be 1 day in length

## Discussion

In our analyses, primary care patients seen during baseline in PROUD trial clinics and non-trial clinics in the same health systems were largely comparable in measured patient demographic and clinical characteristics and baseline measures of main trial outcomes; because the non-trial clinics were randomly selected from those not in the trial (but eligible based on the PROUD trial criteria), we expect this finding to generalize to other similarly-sized eligible clinics in the same health systems. There were a few minor differences in patient characteristics: patients in trial clinics appeared to be slightly younger, more likely to be Hispanic or Latinx, less likely to be white, less likely to have Medicare insurance, more likely to have Medicaid/subsidized insurance, and more likely to live in neighborhoods with higher poverty levels than patients seen in non-trial clinics. There were slightly more primary care providers in the trial clinics than the non-trial clinics, but the number of buprenorphine prescribers were similar. We will take these minor differences into account when considering the generalizability of the PROUD trial’s findings. Importantly, there were no significant differences in the baseline measures of either the implementation outcome (days of OUD medication treatment) or the effectiveness outcome (days of acute care utilization in patients with OUD) between trial and non-trial clinics.

This is the first study, to our knowledge, to compare patients in clinics participating in a pragmatic cluster-randomized trial with patients in other clinics from the same health system that did not participate. Several prior studies compared individual patients participating in trials with eligible non-participants, with conflicting findings [4, 5, 17]. One analysis assessed representativeness in a trial of a psychosocial intervention among patients with severe mental illness and found that participating patients had better mental health status than those who were eligible but declined participation [3]. Another analysis found that among patients undergoing dialysis, those in the general population were older and had different patterns of comorbidities than those who participated in large, multicenter randomized controlled trials [4]. An additional study compared clinical characteristics of participating and non-participating eligible patients in 3 separate trials [5]. Two of the trials were surgical trials that found no difference between participating patients and eligible non-participating patients. However, in the third one (a trial of children with leukemia and lymphoma), participating patients were considerably younger, more likely to be male, more likely to be white, and less likely to use antineoplastic, opioid, and anti-acid medications than eligible non-participants. Olsen, et al. [18], commented on the issue of representativeness in public policy research, noting that studies in education and social services often select sites for recruitment in a non-random way (akin to clinics in PROUD), based on characteristics that may impact the effect of the policy. The authors argued that this non-random selection, combined with some sites opting out of study participation, leads to potential lack of generalizability of the effect estimate even in very pragmatic studies.

This study was subject to limitations. Although we reported information on a variety of patient characteristics and outcomes, certain factors that may have played a large role in the clinics’ willingness and ability to participate in the trial, and could impact success of implementation of the intervention, were not captured in standard electronic health record data, including clinic leadership and provider attitudes regarding medication treatment for OUD. Although some of this information was captured via interviews and surveys with the trial clinics, it was not available for non-trial clinics, and thus was not included in this analysis. Clinics that were selected to participate in the trial likely differed in unmeasured ways from those that were not selected to participate. Additionally, we assessed data in a “snapshot” of the 2-year baseline period instead of assessing patterns of change over time, which could have provided useful information about the trajectory of our outcomes. Most health systems in this analysis were not integrated health systems, meaning they provided health care but not insurance. Thus, when patients received care outside of the health system, we were unable to capture information on their health care utilization, diagnoses, or medication use. Some patients who were eligible based on a primary care visit early in the 3-year eligibility period may have left the health system. Of note, recent specifications of the Massachusetts OBAT model for a Medicaid program required further infrastructure which was not required in PROUD [8]. Clinics in the PROUD trial were recruited in Phase 1 of PROUD by health system lead investigators, who had to identify two comparable, adequately sized clinics in their health system [7]. We did not a require a systematic process of recruitment because such a process might have made the trial infeasible by adding further barriers to participation; as it was, only 6 of 11 health systems were able to identify trial clinics that qualified for participation. As previously noted, to include four non-trial clinics in each health system for this analysis, the inclusion criterion was lowered to ≥7500 patients seen annually for non-trial clinics. Therefore, although the non-trial clinics were smaller than the trial clinics on average, likely accounting for smaller numbers of primary care providers in non-trial clinics, that did not appear to impact their ability to diagnose and treat OUD. Also, even after relaxing this criterion, one health system in the PROUD trial did not have non-trial primary care clinics that were large enough to be included in this analysis. Finally, although the randomized clinics in the PROUD trial appeared to be representative of other clinics in the geographically and organizationally diverse health systems in the trial (in terms of measures in this analysis), they may not be representative of other health systems in the US, and around the world, which did not participate.

Health system electronic data is a recommended resource for pragmatic trials to assess the similarity of randomized and non-randomized samples, and thus generalizability of the findings [19]; a key strength of this analysis was the ability to use electronic health data from health systems (i.e., not primary data collection). This allowed us to collect many of the same data elements on non-trial clinics without substantial additional effort or recruitment biases. Use of these secondary data allowed assessment not only of baseline patient characteristics in trial and non-trial clinics, but also comparison of baseline measures of the main trial outcomes in trial and non-trial clinics.

## Conclusion

Our findings suggest that clinics participating in a pragmatic implementation trial of treatment for OUD in primary care were largely representative, in terms of baseline patient characteristics and baseline measures of trial outcomes, of other primary care clinics of similar size in the same health systems. Although this analysis cannot speak to generalizability more broadly, the findings are valuable for the PROUD trial. Additionally, this analysis can be used as an example for future pragmatic or implementation trials, as well as quality improvement projects, to assess the representativeness of participating clinics.

## Supplementary Information

**Additional file 1.**

## Data Availability

The datasets created and analyzed during the current study are not publicly available because this type of data sharing was not approved in the data use agreements with the participating health systems, and there is not presently funding to modify the data use agreement, obtain Institutional Review Board approval, and de-identify and share the datasets. The datasets are available from the corresponding author on reasonable request including funding for the aforementioned tasks.

## Abbreviations

PROUD trial: Primary care Opioid Use Disorders Treatment trial
OUD: Opioid use disorder
XR-NTX: Extended-release injectable naltrexone
SD: Standard deviation
MD: Mean difference
SMD: Standardized mean difference
