# Supplementary material for: Baseline representativeness of patients in clinics enrolled in the PRimary care Opioid Use Disorders treatment (PROUD) trial: comparison of trial and non-trial clinics in the same health systems
Source: BMC Health Serv Res. 2022 Dec 29;22:1593. doi: 10.1186/s12913-022-08915-1 (PMC9801668; doi:10.1186/s12913-022-08915-1)
Supplement: Supplementary file 1 — Additional file 1. [file 12913_2022_8915_MOESM1_ESM.docx]

**Supplement**

**Supplemental Table 1.** Opioid use disorder (OUD) ICD-9 and ICD-10 diagnosis codes

| **Code Description** | **Code** |
| --- | --- |
| Opioid abuse | F11.1 |
| Opioid abuse, uncomplicated | F11.10 |
| Opioid abuse with intoxication, uncomplicated | F11.120 |
| Opioid abuse with intoxication delirium | F11.121 |
| Opioid abuse with intoxication with perceptual disturbance | F11.122 |
| Opioid abuse with intoxication, unspecified | F11.129 |
| Opioid abuse with opioid-induced mood disorder | F11.14 |
| Opioid abuse with opioid-induced psychotic disorder | F11.15 |
| Opioid abuse with opioid-induced psychotic disorder with delusions | F11.150 |
| Opioid abuse with opioid-induced psychotic disorder with hallucinations | F11.151 |
| Opioid abuse with opioid-induced psychotic disorder, unspecified | F11.159 |
| Opioid abuse with other opioid-induced disorder | F11.18 |
| Opioid abuse with opioid-induced sexual dysfunction | F11.181 |
| Opioid abuse with opioid-induced sleep disorder | F11.182 |
| Opioid abuse with other opioid-induced disorder | F11.188 |
| Opioid abuse with unspecified opioid-induced disorder | F11.19 |
| Opioid dependence, uncomplicated | F11.20 |
| Opioid dependence with intoxication, uncomplicated | F11.220 |
| Opioid dependence with intoxication delirium | F11.221 |
| Opioid dependence with intoxication with perceptual disturbance | F11.222 |
| Opioid dependence with intoxication, unspecified | F11.229 |
| Opioid dependence with withdrawal | F11.23 |
| Opioid dependence with opioid-induced mood disorder | F11.24 |
| Opioid dependence with opioid-induced psychotic disorder with delusions | F11.250 |
| Opioid dependence with opioid-induced psychotic disorder with hallucinations | F11.251 |
| Opioid dependence with opioid-induced psychotic disorder, unspecified | F11.259 |
| Opioid dependence with other opioid-induced disorder | F11.28 |
| Opioid dependence with opioid-induced sexual dysfunction | F11.281 |
| Opioid dependence with opioid-induced sleep disorder | F11.282 |
| Opioid dependence with other opioid-induced disorder | F11.288 |
| Opioid dependence with unspecified opioid-induced disorder | F11.29 |
| Opioid type dependence, unspecified | 304.00 |
| Opioid type dependence, continuous | 304.01 |
| Opioid type dependence, episodic | 304.02 |
| Combinations of opioid type drug with any other drug dependence, unspecified | 304.70 |
| Combinations of opioid type drug with any other drug dependence, continuous | 304.71 |
| Combinations of opioid type drug with any other drug dependence, episodic | 304.72 |
| Opioid abuse, unspecified | 305.50 |
| Opioid abuse, continuous | 305.51 |
| Opioid abuse, episodic | 305.52 |

**Supplemental Table 2.** Procedure codes for buprenorphine formulations for opioid use disorder (OUD) treatment and extended release injectable naltrexone (XR-NTX)

| **Code description** | **Codes** |
| --- | --- |
| Buprenorphine implant | J0570, G2070, G2072 |
| Buprenorphine injection | Q9991, Q9992, G2069 |
| Oral buprenorphine | J0571, G2068, G2079 |
| Oral buprenorphine with naloxone | J0572, J0573, J0574, J0575 |
| Naltrexone injection | J2315, G2073, HZ84ZZZ, HZ94ZZZ |

**Supplemental Table 3.** Fatal and non-fatal opioid overdose, follow up for opioid overdose, and sequelae ICD-10 diagnosis codes

| **Code description** | **Code** |
| --- | --- |
| Accidental poisoning by heroin | E850.0 |
| Accidental poisoning by methadone | E850.1 |
| Accidental poisoning by other opiates and related narcotics | E850.2 |
| Poisoning by, adverse effect of and underdosing of opium | T40.0 |
| Poisoning by, adverse effect of and underdosing of opium | T40.0X |
| Poisoning by opium, accidental (unintentional) | T40.0X1 |
| Poisoning by opium, accidental (unintentional), initial encounter | T40.0X1A |
| Poisoning by opium, accidental (unintentional), subsequent encounter | T40.0X1D |
| Poisoning by opium, accidental (unintentional), sequela | T40.0X1S |
| Poisoning by opium, intentional self-harm | T40.0X2 |
| Poisoning by opium, intentional self-harm, initial encounter | T40.0X2A |
| Poisoning by opium, intentional self-harm, subsequent encounter | T40.0X2D |
| Poisoning by opium, intentional self-harm, sequela | T40.0X2S |
| Poisoning by opium, assault | T40.0X3 |
| Poisoning by opium, assault, initial encounter | T40.0X3A |
| Poisoning by opium, assault, subsequent encounter | T40.0X3D |
| Poisoning by opium, assault, sequela | T40.0X3S |
| Poisoning by opium, undetermined | T40.0X4 |
| Poisoning by opium, undetermined, initial encounter | T40.0X4A |
| Poisoning by opium, undetermined, subsequent encounter | T40.0X4D |
| Poisoning by opium, undetermined, sequela | T40.0X4S |
| Poisoning by and adverse effect of heroin | T40.1 |
| Poisoning by and adverse effect of heroin | T40.1X |
| Poisoning by heroin, accidental (unintentional) | T40.1X1 |
| Poisoning by heroin, accidental (unintentional), initial encounter | T40.1X1A |
| Poisoning by heroin, accidental (unintentional), subsequent encounter | T40.1X1D |
| Poisoning by heroin, accidental (unintentional), sequela | T40.1X1S |
| Poisoning by heroin, intentional self-harm | T40.1X2 |
| Poisoning by heroin, intentional self-harm, initial encounter | T40.1X2A |
| Poisoning by heroin, intentional self-harm, subsequent encounter | T40.1X2D |
| Poisoning by heroin, intentional self-harm, sequela | T40.1X2S |
| Poisoning by heroin, assault | T40.1X3 |
| Poisoning by heroin, assault, initial encounter | T40.1X3A |
| Poisoning by heroin, assault, subsequent encounter | T40.1X3D |
| Poisoning by heroin, assault, sequela | T40.1X3S |
| Poisoning by heroin, undetermined | T40.1X4 |
| Poisoning by heroin, undetermined, initial encounter | T40.1X4A |
| Poisoning by heroin, undetermined, subsequent encounter | T40.1X4D |
| Poisoning by heroin, undetermined, sequela | T40.1X4S |
| Poisoning by, adverse effect of and underdosing of other opioids | T40.2 |
| Poisoning by, adverse effect of and underdosing of other opioids | T40.2X |
| Poisoning by other opioids, accidental (unintentional) | T40.2X1 |
| Poisoning by other opioids, accidental (unintentional), initial encounter | T40.2X1A |
| Poisoning by other opioids, accidental (unintentional), subsequent encounter | T40.2X1D |
| Poisoning by other opioids, accidental (unintentional), sequela | T40.2X1S |
| Poisoning by other opioids, intentional self-harm | T40.2X2 |
| Poisoning by other opioids, intentional self-harm, initial encounter | T40.2X2A |
| Poisoning by other opioids, intentional self-harm, subsequent encounter | T40.2X2D |
| Poisoning by other opioids, intentional self-harm, sequela | T40.2X2S |
| Poisoning by other opioids, assault | T40.2X3 |
| Poisoning by other opioids, assault, initial encounter | T40.2X3A |
| Poisoning by other opioids, assault, subsequent encounter | T40.2X3D |
| Poisoning by other opioids, assault, sequela | T40.2X3S |
| Poisoning by other opioids, undetermined | T40.2X4 |
| Poisoning by other opioids, undetermined, initial encounter | T40.2X4A |
| Poisoning by other opioids, undetermined, subsequent encounter | T40.2X4D |
| Poisoning by other opioids, undetermined, sequela | T40.2X4S |
| Poisoning by methadone, accidental (unintentional) | T40.3X1 |
| Poisoning by methadone, accidental (unintentional), initial encounter | T40.3X1A |
| Poisoning by methadone, accidental (unintentional), subsequent encounter | T40.3X1D |
| Poisoning by methadone, accidental (unintentional), sequela | T40.3X1S |
| Poisoning by methadone, intentional self-harm | T40.3X2 |
| Poisoning by methadone, intentional self-harm, initial encounter | T40.3X2A |
| Poisoning by methadone, intentional self-harm, subsequent encounter | T40.3X2D |
| Poisoning by methadone, intentional self-harm, sequela | T40.3X2S |
| Poisoning by methadone, assault | T40.3X3 |
| Poisoning by methadone, assault, initial encounter | T40.3X3A |
| Poisoning by methadone, assault, subsequent encounter | T40.3X3D |
| Poisoning by methadone, assault, sequela | T40.3X3S |
| Poisoning by methadone, undetermined | T40.3X4 |
| Poisoning by methadone, undetermined, initial encounter | T40.3X4A |
| Poisoning by methadone, undetermined, subsequent encounter | T40.3X4D |
| Poisoning by methadone, undetermined, sequela | T40.3X4S |
| Poisoning by other synthetic narcotics, accidental (unintentional) | T40.4X1 |
| Poisoning by other synthetic narcotics, accidental (unintentional), initial encounter | T40.4X1A |
| Poisoning by other synthetic narcotics, accidental (unintentional), subsequent encounter | T40.4X1D |
| Poisoning by other synthetic narcotics, accidental (unintentional), sequela | T40.4X1S |
| Poisoning by other synthetic narcotics, intentional self-harm | T40.4X2 |
| Poisoning by other synthetic narcotics, intentional self-harm, initial encounter | T40.4X2A |
| Poisoning by other synthetic narcotics, intentional self-harm, subsequent encounter | T40.4X2D |
| Poisoning by other synthetic narcotics, intentional self-harm, sequela | T40.4X2S |
| Poisoning by other synthetic narcotics, assault | T40.4X3 |
| Poisoning by other synthetic narcotics, assault, initial encounter | T40.4X3A |
| Poisoning by other synthetic narcotics, assault, subsequent encounter | T40.4X3D |
| Poisoning by other synthetic narcotics, assault, sequela | T40.4X3S |
| Poisoning by other synthetic narcotics, undetermined | T40.4X4 |
| Poisoning by other synthetic narcotics, undetermined, initial encounter | T40.4X4A |
| Poisoning by other synthetic narcotics, undetermined, subsequent encounter | T40.4X4D |
| Poisoning by other synthetic narcotics, undetermined, sequela | T40.4X4S |
